# Standardizing Care for Agitation in Alzheimer’ Dementia: Results from a Randomized Controlled Trial of an Integrated Care Pathway versus Usual Care ‐The StaN trial

**DOI:** 10.1002/alz.093091

**Published:** 2025-01-03

**Authors:** Sanjeev Kumar, Amer M. Burhan, Li Chu, Sarah Colman, Simon Davies, Peter Derkach, Sarah Elmi, Philip Gerretsen, Ariel Graff‐Guerrero, Maria Hussain, Zahinoor Ismail, Donna Kim, Linda Krisman, Clement Ma, Rola Moghabghab, Benoit H. Mulsant, Vasavan Nair, Bruce G. Pollock, Soham Rej, Aviva Rostas, David Streiner, Lisa Van Bussel, Tarek K. Rajji, StaN Study Group

**Affiliations:** ^1^ Temerty Faculty of Medicine, University of Toronto, Toronto, ON Canada; ^2^ Centre for Addiction and Mental Health, Toronto, ON Canada; ^3^ Toronto Dementia Research Alliance, Toronto, ON Canada; ^4^ University of Toronto, Toronto, ON Canada; ^5^ Ontario Shores Centre for Mental Health Sciences, Whitby, ON Canada; ^6^ Ukrainian Canadian Care Centre, Toronto, ON Canada; ^7^ West Park Health Care Centre, Toronto, ON Canada; ^8^ Department of Psychiatry, Temerty Faculty of Medicine, University of Toronto, Toronto, ON Canada; ^9^ Queen’s University, Kingston, ON Canada; ^10^ Hotchkiss Brain Institute, University of Calgary, Calgary, AB Canada; ^11^ Division of Biostatistics, Dalla Lana School of Public HealthUniversity of Toronto, Toronto, ON Canada; ^12^ McGill University, Montreal, QC Canada; ^13^ McMaster University, Hamilton, ON Canada; ^14^ Western University, London, ON Canada; ^15^ Temerty Faculty of Medicine, Division of Psychiatry, University of Toronto, Toronto, ON Canada; ^16^ Toronto Dementia Research Alliance, University of Toronto, Toronto, ON Canada

## Abstract

**Background:**

Adherence to treatment guidelines for agitation in dementia is suboptimal and inconsistent. We designed an Integrated Care Pathway (ICP) that standardized behavioral and pharmacological interventions for agitation in dementia, and evaluated it against treatment‐as‐usual (TAU). The two primary hypotheses were that, compared to TAU, the ICP would result in (1) lower agitation and (2) lower rates of polypharmacy at study end.

**Method:**

The Standardizing Care for Neuropsychiatric Symptoms and Quality of Life in Dementia (StaN) trial (**ClinicalTrials.gov #** NCT03672201) was conducted at five academically affiliated inpatient units (Inpatient) and seven long‐term‐care homes (LTCHs). Participants with agitation related to Alzheimer’s dementia were randomized 1:1 to receive the ICP or TAU for 12 weeks. Primary outcomes were: (1) Cohen Mansfield Agitation Inventory (CMAI) completed at weeks 3, 8, and 12 (primary), and (2) polypharmacy defined as using more than one psychotropic medication assessed at weeks 1, 3, 4, 6, 8, 10, and 12 (primary) post‐randomization. Linear mixed effect models and generalized estimating equations were used to test our hypotheses controlling for age, gender, and stage of dementia. The study was powered for Inpatient and LTCH settings separately.

**Result:**

185 participants were randomized: 93 in Inpatient (46 ICP: 47 TAU; females = 32 (34.4%); mean (standard deviation [SD]) age = 75.0 (8.4) years), and 92 in LTCH (46 ICP: 46 TAU; women = 63 (68.5%); mean (SD) age = 85.9 (7.6) years). There were no significant time*group interactions for the CMAI scores for Inpatient (F_4, 297.9_ = 0.8, p = 0.53) or LTCH (F_4, 297.3_ = 1.1, p = 0.36) and no significant differences at week‐12 (Inpatient: ICP‐TAU adjusted difference = 0.025; 95% Confidence Interval (CI): ‐0.410, 0.460; LTCH: ICP‐TAU adjusted difference = ‐0.214; 95%CI: ‐0.699, 0.270). However, there were significant time*group interactions for polypharmacy for both Inpatient (χ^2^_7_ = 18.6, p = 0.01) and LTCH (Χ^2^_7_ = 22.9, p = 0.002). Differences were not significant at week‐12 (Inpatient: ICP‐TAU adjusted difference = 0.15; 95%CI: ‐0.11, 0.40; LTCH: ICP‐TAU adjusted difference = 0.33; 95%CI: ‐0.06, 0.72), the ICP group had lower rates of polypharmacy than TAU group at weeks 3, 4, and 6 on Inpatient, and week 3 in LTCH.

**Conclusion:**

Standardizing care for agitation in dementia may result in less polypharmacy without affecting efficacy. Future studies should assess the ICP in broader community and outpatient settings.

